# Comprehensive bibliometric research in neuroscience: focusing on ophthalmology

**DOI:** 10.3389/fnins.2023.1106023

**Published:** 2023-06-15

**Authors:** Xiaojing Xia, Lijun Li, Zeyu Cheng, Qiyu Chen, Tao Huang, Yun Yu, Lei Shang

**Affiliations:** Jiangxi Clinical Research Center for Ophthalmic Disease, Jiangxi Research Institute of Ophthalmology and Visual Science, Affiliated Eye Hospital of Nanchang University, Nanchang, China

**Keywords:** neuroscience, bibliometric, visualization, ophthalmology, eye tracking

## Abstract

**Background:**

This study aimed to comprehensively summarize the knowledge structure and research hotspots of ophthalmology in the field of neuroscience through bibliometric and visual analysis.

**Methods:**

We searched the Web of Science Core Collection database for articles from 2002 to 2021 related to ophthalmology in the field of neuroscience. Using VOSviewer and CiteSpace, bibliometric analysis was conducted on the number of annual ophthalmology publications, authors, organizations, countries, journals, cited references, keywords, and burst keywords.

**Results:**

A total of 9,179 articles were published from 34,073 authors, 4,987 organizations, and 87 countries. The cited references in these articles were published in 23,054 journals. Moreover, there were 30,864 keywords among the 9,179 articles. Notably, scholars have increasingly begun paying attention to ophthalmology in the field of neuroscience in the past 20 years. Claudio Babiloni published the most articles. The University of Washington had the greatest number of articles. The United States, Germany, and England led in the number of articles published. The Journal of Neuroscience was the most cited. The article with the highest outbreak intensity was an article published by Maurizio Corbetta in Nature Reviews Neuroscience in 2002 entitled “Control of goal-directed and stimulus-driven attention in the brain.” The most important keyword was the brain, and the top burst keyword was functional connectivity.

**Conclusion:**

This study visualized ophthalmology research in the field of neuroscience through bibliometric analysis and predicted potential research trends in future to help clinicians and basic researchers provide diversified perspectives and further carry out in-depth research on ophthalmology.

## 1. Introduction

Ophthalmology is a discipline of medicine that deals with the study of diseases of the visual systems, including the eyeball and its associated tissues (Cursiefen, 2019). Conversely, neuro-ophthalmology is a new inter-disciplinary branch of ophthalmology, neurology, and neurosurgery (Wei, 2014; Wei et al., 2020; Cherayil and Tamhankar, 2021). Neuro-ophthalmic diseases encompass those that affect all regions studied under neuro-ophthalmology; these are mainly neurological diseases related to eye perception and movement (Clark and Eggenberger, 2012). Depending on the location and type of lesions, these diseases may simply manifest as vision loss, diplopia, complex syndromes, or systemic diseases. If not diagnosed and treated in time, these may lead to permanent vision loss or other significant diseases, or even death (Duong et al., 2008; Spiegel and Moss, 2021).

Scientists have associated neuroscience with ophthalmology; studies have revealed that 10% of all brain injuries cause symptoms of eye disease, such as cortical blindness, hemiopia, and scotoma (Brown and Harvey, 1941; Fishman, 1995). Hence, scholars have been prompted to perform further research to improve our understanding of the relationship between ophthalmology and the nervous system. Thus, discoveries from a variety of research fields (including machine vision, visual psychophysics, visual neurophysiology, developmental neurobiology, brain imaging, and joint electrophysiological) as well as psychophysical studies in behavioral primates are beginning to establish a causal relationship between neural activity and visual perception (Yao et al., 2010; Norcia, 2013; Roussy et al., 2021). In clinical trials, experts continue to improve and summarize the diagnosis and treatment of ophthalmic diseases in the field of neuroscience (Neuro-ophthalmology Group of Ophthalmology Branch of Chinese Medical Association, 2022). Furthermore, with the development of machine learning, artificial intelligence is gradually being applied to ophthalmology; originally considered for retinal diseases and glaucoma, its application in ophthalmology was explored, and its potential for assisting with vision care and improving clinical diagnosis and prediction was discovered gradually (Rampat et al., 2021). Cornelissen et al. used functional magnetic resonance imaging in combination with model-driven analyses to quantify changes in cortical functional organization in participants with glaucoma (Carvalho et al., 2022). The co-registration of signals and eye movements was used to obtain real-time data on brain activity (Ionescu et al., 2022). Moreover, considerable ophthalmology-related basic research on the nervous system has also attracted the attention of scholars. Jay Hegdé systematically discussed the current understanding of the neural mechanisms of advanced vision (Hegde, 2018). Yau et al. focused on the flow of molecular signals that are important to sight and smell (Chai et al., 2020; Chen et al., 2023). Xiong et al. focused on retinal neuronal death caused by ischemia–reperfusion injury (Wan et al., 2023; Yan et al., 2023; Zhang et al., 2023; Zhou et al., 2023).

With the development of computer science and informatics, bibliometric analysis can provide a new perspective for sorting the development and research trends of a certain field (in this case, ophthalmology in the field of neuroscience). Bibliometric analysis is a quantitative literature analysis methodology that involves obtaining key information from publications, such as authors, journals, institutions, references, and keywords, with the help of modern scientific and technological research tools [such as VOSviewer (Waltman et al., 2010) and CiteSpace (Chen, 2006)]. It mines the internal relationships among the obtained data, which improve the understanding of progress in the studied frontier (van Eck and Waltman, 2010; Mayr and Scharnhorst, 2015; Chen, 2017; Chen and Song, 2019). Guo et al. conducted a bibliometric analysis of the top 100 cited articles on pediatric ophthalmology to explore their distribution in terms of the research categories, journals, and institutions (Oydanich et al., 2022). Mouriaux analyzed literature on ocular tumors published between 1966 and 2012 (Boudry and Mouriaux, 2015). Medawar et al. analyzed the bibliometric trends of ophthalmology from 1997 to 2009 (Mansour et al., 2015). Liu et al. conducted a bibliometric analysis of ophthalmology hotspots and trends from 2017 to 2021 (Tan et al., 2022). These scholars focused on summarizing the progress of research in ophthalmology through bibliometric methods. However, bibliometric research in ophthalmology in the field of neuroscience remains in a vacuum. Therefore, this study used bibliometrics to summarize the occurrence and development trends of ophthalmology in neuroscience in the past two decades based on the Web of Science Core Collection, which is a global authority and comprehensive citation index database. Our findings may provide suggestions to researchers in this field or offer insights into finding new research hotspots in neuroscience.

## 2. Methods

### 2.1. Data sources

**Figure 1:**
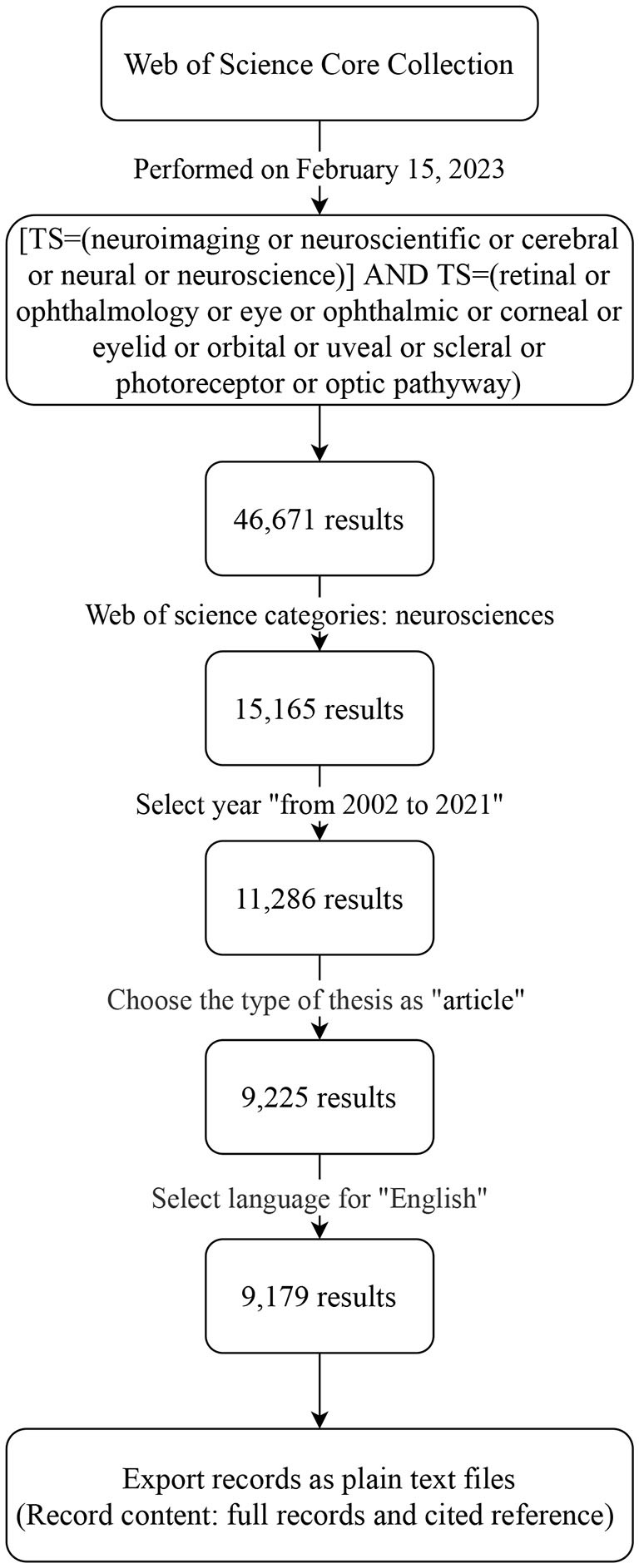
Data source and research strategy.

Literature data for this bibliometrics study were retrieved from the Web of Science Core Collection (Falagas et al., 2008). The selected index topic was [Topic Search (TS) = (neuroimaging or neuroscientific or cerebral or neural or neuroscience)] AND TS = (retinal or ophthalmology or eye or ophthalmic or corneal or eyelid or orbital or uveal or scleral or photoreceptor or optic pathway). Due to technical limitations, the keywords of this article were not full-text search but selected and analyzed from the title, the abstract, and keywords. The Web of Science category was as follows: neurosciences. The other parameters were set as follows: data range, “from 2002 to 2021”; document type, “article” (no reviews, patents, abstracts, and papers were included); and language, English. All data were collected on 15 February 2023. Then, we exported the full record as a plain text file and cited references for further research (Figure 1).

### 2.2. Data analysis

According to the obtained files, VOSviewer_1.6.19^1^ and CiteSpace 6.1.R6 (64-bit)^2^ were used to create a visual map from the obtained files to analyze the distribution of authors, organizations, countries, journals, references, and keywords related to ophthalmology in the field of neuroscience; these were ranked using Microsoft Excel.^3^

#### 2.2.1. Analysis of annual publications

The “heat” of a research field is indicated by the number of articles published in that field. The number of articles published annually was determined chronologically, and a histogram of the number of published articles was obtained. Then, the distribution of the number of published articles was displayed by a linear-fitting trend line, where *X* represented the year of the relative base period (which for these articles was 2001) and Y represented the number of articles published per year.

#### 2.2.2. Co-authorship analysis

Co-authorship analysis is a type of cooperation analysis and was performed using VOSviewer. The purpose was to understand the cooperation among authors, organizations, and countries in the field. First, we selectively created a map based on bibliographic data and then selected read data from bibliographic files using supported file types: We imported data from the Web of Science and then selected the type of analysis as “co-authorship”. Finally, we selected the corresponding research content in the unit of analysis, set the threshold according to the purpose of the display and obtained the corresponding graph, and exported the rankings displayed by the graph. The number and thickness of the lines in the graph indicate the closeness and strength of the relationship, respectively. The color represents the average publication year, which is displayed in the lower right corner of the figure. The size of the frame or circle represents the degree of contribution or influence.

#### 2.2.3. Co-citation cited analysis

Co-citation analysis is a combination analysis performed using VOSviewer and CiteSpace. The purpose was to master the studies that are frequently cited in a research field as well as the journals that publish these articles. The VOSviewer analysis method used here was the same as that described in Section 2.2.2, but the type of analysis selected was “co-citation”. Thereafter, we selected the corresponding research content in the unit of analysis and finally set the threshold according to the purpose displayed; we then obtained the corresponding graph and ranking. Each node represents a cited article, and the connection between the two nodes represents the cited relationship. The size of the frame or circle indicates the contribution or influence of the cited references. Conversely, the CiteSpace analysis required data to be pioneered into a format that the software could analyze further. First, duplicates in the obtained files were removed by selecting “Import/Export” from the “Data” menu; the exported files were further analyzed. In the control panel of the software, time slicing was performed to obtain 1-year slices from January 2002 to December 2021. In the selection criteria, the value of the scale factor *k* was adjusted (*k* = 10) in the *g*-index. The *g*-index is the largest number that equals the average number of citations of the most highly cited *g* publications (Egghe, 2006). Finally, we selected the corresponding research content in the node type for analysis by conducting a burst detection. Burst detection has two attributes, namely burst intensity and burst duration; these can be used for the following purposes: (1) to detect instances of great changes in the amounts of citations in a certain period of time and (2) to identify a decline or rise in the cited literature or keywords (Zhu et al., 2020).

#### 2.2.4. Keyword and burst keyword analysis

The frequency and centrality of keywords were analyzed using a combination of VOSviewer and CiteSpace, and the methodology was the same as that described in Sections 2.2.2 and 2.2.3.

## 3. Results

### 3.1. Number of annual publications

**Figure 2:**
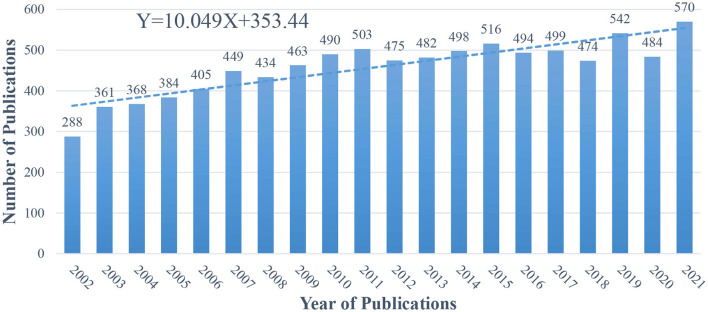
Number of annual publications from 2002 to 2021 related to ophthalmology in the field of neuroscience.

In the last 20 years, 9,179 articles were published on ophthalmology in the field of neuroscience, with an average of 458.95 articles published each year. In terms of annual publications, the year 2002 (*n* = 288) had the fewest articles and the year 2021 (*n* = 570) had the most. The distribution of the number of published articles was linearly fitted with the trend line *Y* = 10.049*X* + 353.44; this finding indicated that article publication in this field exhibited a steady upward trend overall (Figure 2).

### 3.2. Co-authorship with respect to authors

**Figure 3:**
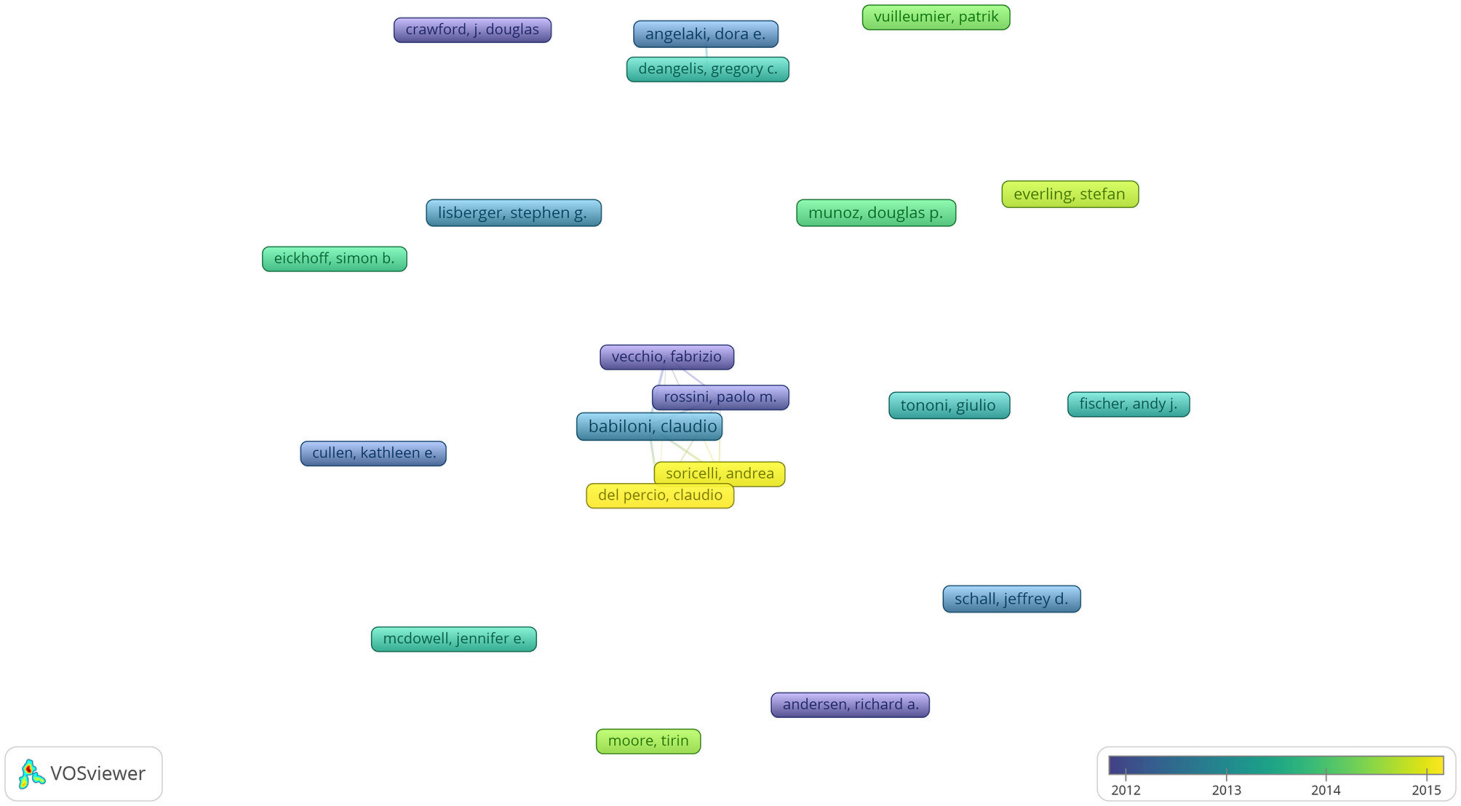
Co-authorship with respect to authors from 2002 to 2021 related to ophthalmology in the field of neuroscience.

The aforementioned 9,179 articles were published by 34,073 authors, with an average of four researchers per article. Using VOSviewer, we analyzed the cooperation among the authors. Figure 3 lists authors with a minimum of 15 published articles; 20 authors reached the threshold. Table 1 shows the top 10 core authors who published at least 18 or more articles in the last 20 years; most of these authors were from Canada and the United States, followed by Italy. Among these authors, Babiloni had the most number of published articles (*n* = 33), followed by Munoz and Schall (*n* = 27 each). Conversely, Tononi ranked first in terms of total citations (*n* = 2,130; averaging 101 citations per article), while Schall ranked second (*n* = 1,350; averaging 50 citations per article). All of these authors contributed to research on ophthalmology in the field of neuroscience. As shown in Figure 3, the intimacy of academic cooperation among the authors is different. The two academic groups are relatively closely connected; one group has five authors with Claudio Babiloni as the core, while the other only has two authors. This suggests that the cooperation among the authors is not closely linked, and further cooperation is needed in future.

**Table 1:** Top 10 co-authorships with respect to authors from 2002 to 2021.

**Rank**	**Authors**	**Affiliations country**	**Organization**	**Documents**	**Citations**	**Average cited/publications**
1	Claudio Babiloni	Italy	Sapienza University of Rome	33	1,340	41
2	Douglas P. Munoz	Canada	Queen's University	27	849	31
3	Jeffrey D. Schall	USA/Canada	Vanderbilt University/York University	27	1,350	50
4	Stephen G. Lisberger	USA	Duke University School of Medicine	26	703	27
5	Dora E. Angelaki	USA	New York University	23	1,101	48
6	Stefan Everling	Canada	The University of Western Ontario	23	692	30
7	Giulio Tononi	USA	University of Wisconsin-Madison	21	2,130	101
8	J. Douglas Crawford	Canada	York University	19	473	25
9	Fabrizio Vecchio	Italy	eCampus University	19	650	34
10	Jennifer E. McDowell	USA	University of Georgia	18	389	22

### 3.3. Co-authorship with respect to organizations

**Figure 4:**
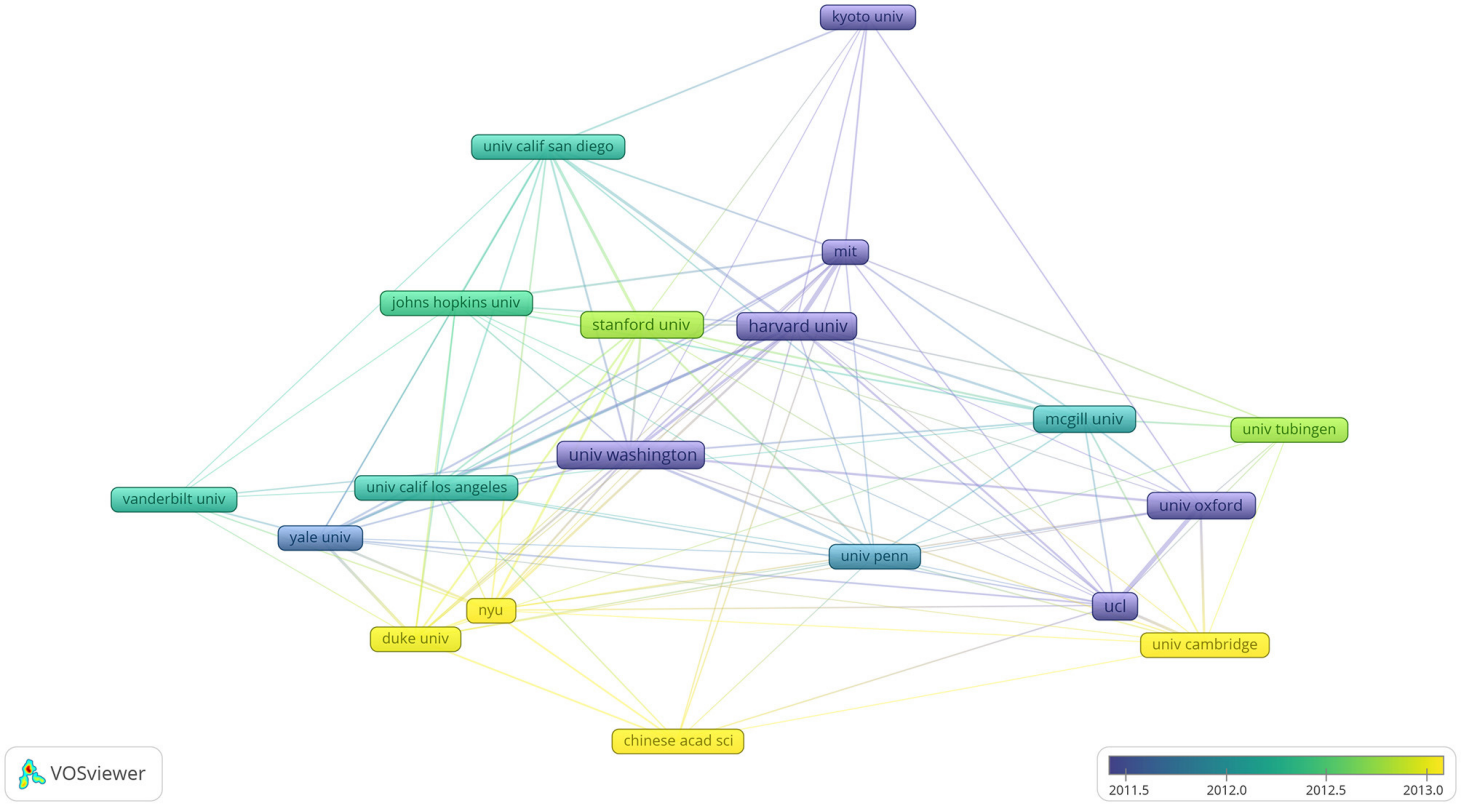
Co-authorship with respect to organizations from 2002 to 2021 related to ophthalmology in the field of neuroscience.

In the last 20 years, 4,987 organizations engaged in neuroscience research in ophthalmology. We used VOSviewer to analyze the citation networks among these organizations. Among organizations with a minimum of 100 published articles, 19 organizations reached the threshold. Figure 4 shows the visualized network of relationships among these organizations, and Table 2 lists the top 10 co-authorship organizations that published the most articles. The most prolific university was the University of Washington (*n* = 250), followed by Harvard University (*n* = 236) and the University College London (*n* = 212). Furthermore, six of the top ten organizations are from the United States, while the remaining four are from the UK and Canada. Harvard University had the highest total citations (*n* = 24,579). Notably, Stanford University ranked fifth in the number of publications (*n* = 145; highest average citations per article: 132). As the hub of basic and cutting-edge scientific research, universities had a high overall publication volume and high citation rate, indicating that the efforts of scholars in these universities made great contributions to the field.

**Table 2:** Top 10 co-authorships with respect to organizations from 2002 to 2021.

**Rank**	**Organization**	**Documents**	**Citations**	**Country**	**Average cited/publications**
1	University of Washington	250	17,680	USA	71
2	Harvard University	236	24,579	USA	104
3	University College London	212	13,463	UK	64
4	McGill University	152	7,693	Canada	51
5	Stanford University	145	19,170	USA	132
6	University of Oxford	145	6,953	UK	48
7	University of California, San Diego	134	7,345	USA	55
8	New York University	129	6,044	USA	47
9	University of Cambridge	114	5,455	UK	48
10	Johns Hopkins University	113	6,295	USA	56

### 3.4. Co-authorship with respect to countries

**Figure 5:**
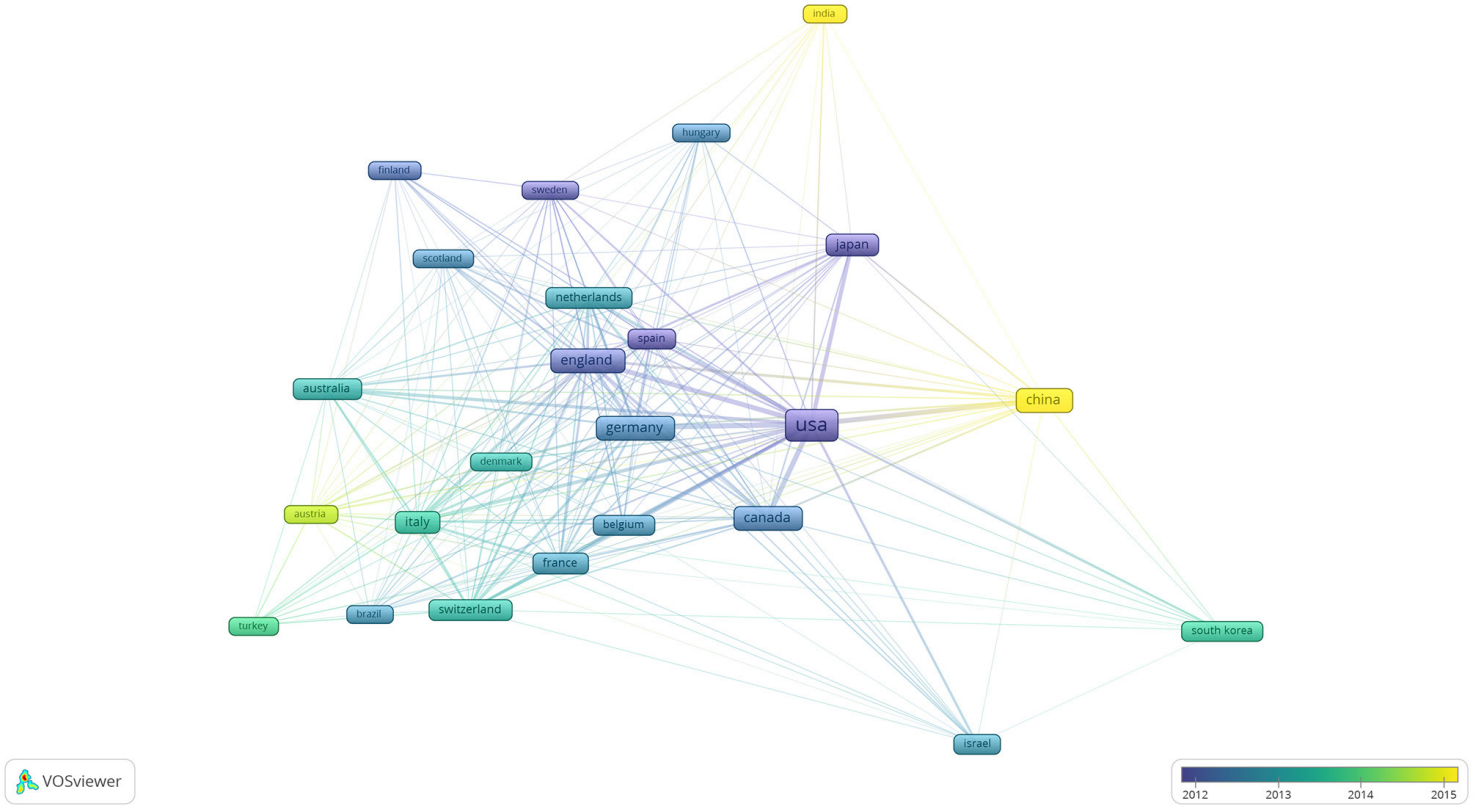
Co-authorship with respect to countries from 2002 to 2021 related to ophthalmology in the field of neuroscience.

In the last 20 years, 87 national research teams were involved in the development of ophthalmology in the field of neuroscience. We used VOSviewer to observe which countries contributed the most to the field. Among the countries with at least 60 published articles, 24 countries reached the threshold. Figure 5 shows the countries that contributed to the field; China and India carried out ophthalmic research later than other countries did, starting around 2015. This indicates that the exploration of ophthalmology neuroscience in these countries is still in its infancy. Table 3 lists the 10 countries with the most publications; these countries have contributed 10,087 articles, which is higher than the total number of articles that were identified (*n* = 9,179). This may be because of the way articles are presented and collected by VOSviewer; one article may be authored by multiple authors from different countries, or one author may be affiliated with different multinational units (for example, Schall, who has conducted research in universities in both the United States and Canada). Among the 10 countries with the highest productivity, the United States has the largest number of publications (*n* = 4,042) and the highest average citations per article (total citations: 215,776; average citations per article: 53). Germany has the second largest number of publications (*n* = 1,047), with 45,260 citations (average citations per article: 43). Finally, England has the third largest number of publications (*n* = 931), with 46,818 citations (average citations per article: 50).

**Table 3:** Top 10 co-authorships with respect to countries from 2002 to 2021.

**Rank**	**Country**	**Documents**	**Citations**	**Total link strength**	**Average cited/publications**
1	USA	4,042	215,776	1,845	53
2	Germany	1,047	45,260	864	43
3	England	931	46,818	933	50
4	China	805	15,700	386	20
5	Canada	771	31,628	535	41
6	Japan	762	22,887	316	30
7	Italy	538	20,095	532	37
8	France	473	18,674	452	39
9	Australia	362	12,174	306	34
10	Netherlands	356	15,755	408	44

### 3.5. Co-citation cited sources and references

**Figure 6:**
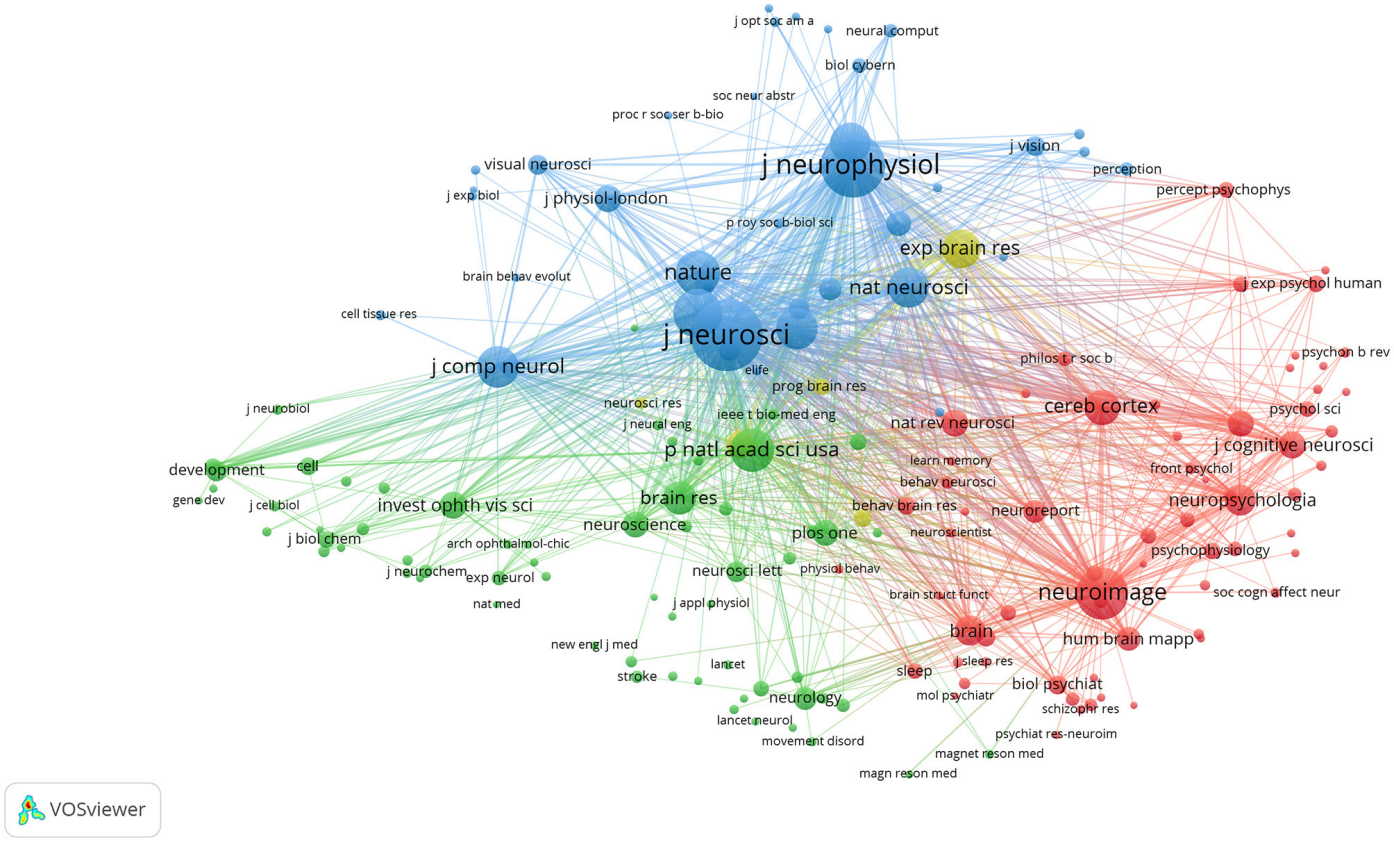
Co-citation cited sources from 2002 to 2021 related to ophthalmology in the field of neuroscience.

The aforementioned 9,179 articles were cited in 23,054 journals; 170 of these journals reached the threshold for at least 400 articles (Figure 6). The Journal of Neuroscience had the highest citation rate (*n* = 33,323), followed by the Journal of Neurophysiology and NeuroImage. The impact factors of these journals were between 1.984 and 69.504 in 2021. The journal Nature had the highest impact factor in 2021, while the journal Vision Research had the lowest (Table 4).

**Table 4:** Top 10 co-citation cited sources from 2002 to 2021.

**Rank**	**Source**	**Citations**	**Total link strength**	**IF (2021)**
1	Journal of Neuroscience	33,323	1,328,330	6.709
2	Journal of Neurophysiology	27,262	1,032,498	2.974
3	NeuroImage	17,292	626,455	7.400
4	Neuron	15,406	679,562	18.688
5	Nature	12,784	543,141	69.504
6	Proceedings of the National Academy of Sciences of the United States of America	12,422	501,377	12.779
7	Science	11,302	481,226	63.714
8	Journal of Comparative Neurology	11,064	453,137	3.028
9	Nature Neuroscience	10,619	479,431	28.771
10	Vision Research	10,596	378,332	1.984

**Figure 7:**
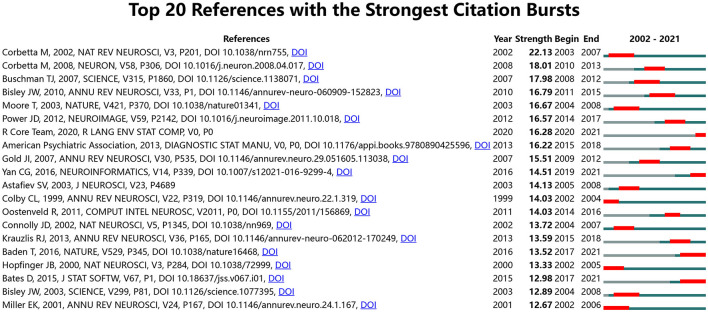
Top 20 references with the strongest citation bursts.

We used CiteSpace to show citation burst references, and Figure 7 lists the top 20 references; Corbetta and Bisley have each authored two of these 20 articles. The article with the highest outbreak intensity was by Corbetta, titled “Control of goal-directed and stimulus-driven attention in the brain (Corbetta and Shulman, 2002),” which was published in Nature Reviews Neuroscience in 2002. The outbreak intensity was 22.13, and the period of burst was mainly from 2003 to 2007. Corbetta also co-authored another article that was published in Neuron in 2008, titled “The reorienting system of the human brain: from environment to theory of mind (Corbetta et al., 2008).” The outbreak intensity was 18.01, and the period of burst was mainly from 2010 to 2013. Bisley published an article titled “Attention, intention, and priority in the parietal lobe (Bisley and Goldberg, 2010)” in the Annual Review of Neuroscience in 2010. The outbreak intensity was 16.79, and the period of burst was mainly from 2011 to 2015. Bisley also co-authored another article that was published in Science in 2003, titled “Neuronal activity in the lateral intraparietal area and spatial attention (Bisley and Goldberg, 2003).” The intensity was 12.89, and the period of burst was mainly from 2004 to 2008. A co-citation analysis of cited sources and references revealed the fundamental studies and the most influential articles in the field. The findings indicated that scholars around the world are constantly deepening their own research fields, expanding their influence, and constantly moving toward high-level fields and inspiring other scholars.

### 3.6. Keyword and burst keywords

**Figure 8:**
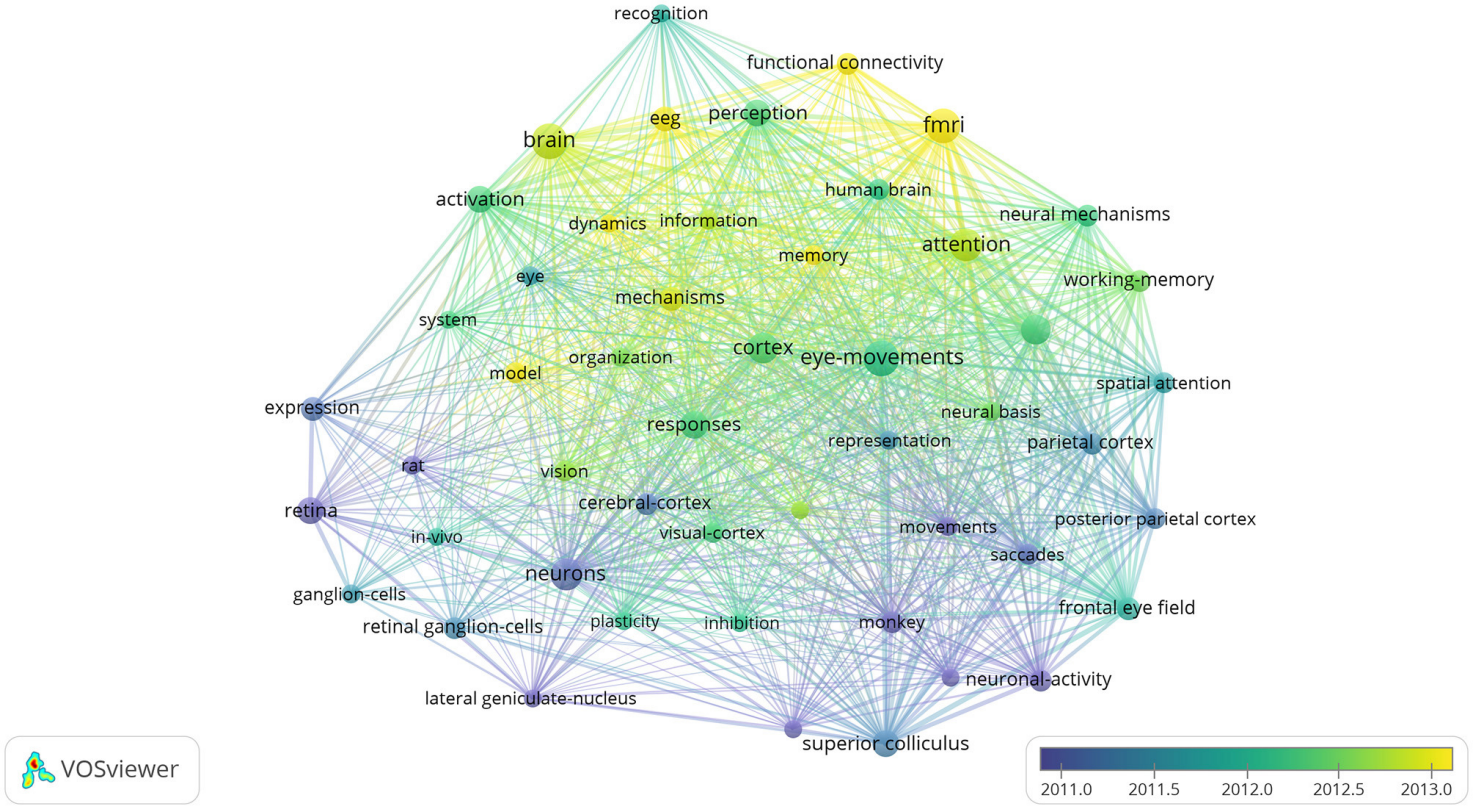
Keywords from 2002 to 2021 related to ophthalmology in the field of neuroscience.

**Figure 9:**
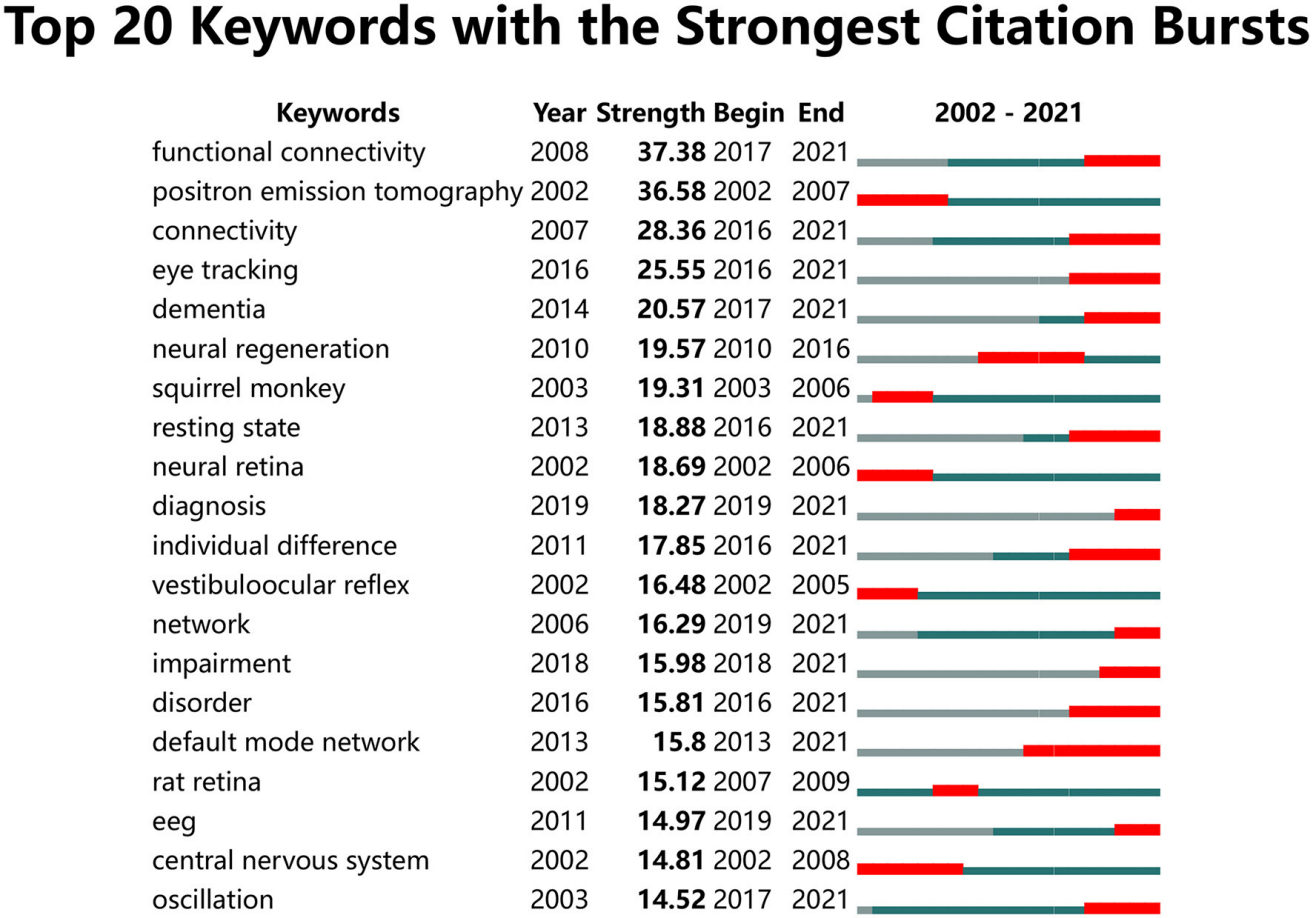
Top 20 keywords with the strongest citation bursts.

A total of 30,864 keywords were retrieved from the aforementioned 9,179 articles. To highlight important keywords, we set the minimum number of occurrences for a keyword to 200, and 50 keywords reached the threshold. The visual network diagram shows the co-occurrence of these keywords, and the high-frequency keywords were “brain,” “eye-movements,” and “fmri” (Figure 8). These keywords were mainly condensed from 2011 to 2013, and “brain” was the most important of all keywords; “cortex” was found to be at the center of the research. Through keyword clustering analysis, three clusters were established, namely neurons, eye movements, and brain imaging. Figure 9 shows the 20 keywords with the highest burst intensity. “Functional connectivity” continued to erupt from 2017 to 2021, with the highest outbreak intensity at 37.38. “Default mode network” received the longest attention, with an outbreak intensity of 15.8 between 2013 and 2021.

## 4. Discussion

In this study, we quantified research on ophthalmology in neuroscience undertaken from 2002 to 2021 using a bibliometric analysis. A total of 9,179 articles were published in the field, and these articles were from 34,073 authors, 4,987 organizations, and 87 countries. The references cited in these articles were published in 23,054 journals, and 30,864 keywords were identified.

In the last two decades, the number of publications on ophthalmology in the field of neuroscience has remained relatively high and showed an upward trend; this indicates that neuro-ophthalmology has attracted the attention of many scholars, who have worked hard and performed in-depth studies in the field. However, ophthalmology belongs to neuroscience, a branch of brain science, and is a relatively young field. The research gaps in this field still need to be identified and addressed.

In the field of neuroscience, studies of the brain were initiated earlier than ophthalmological studies; in fact, the brain was first mentioned in the 17th century BC in the Edwin Smith surgical papyrus to describe the symptoms, diagnosis, and prognosis of two patients with head injuries (Schultz, 2001). Since then, brain research has undergone philosophical, experimental, and theoretical phases (Fan and Markram, 2019). Scientists dissect the brain to understand its structure and function (Di Ieva et al., 2007). Developments in science and technology have enabled in-depth studies on single molecules and cells to the brain sensory, motor, and cognitive and imaging in neuroscience (Kandel et al., 2013; Shulman, 2013). In ancient Greece, Alcmaeon dissected the eyes and linked the brain to vision after an analysis. This presented an opportunity for subsequent researchers to undertake ophthalmological studies (Gross, 2009). Thus, neuroscientists continued to deepen research and development. In 1899, Francis Gotch began to pay attention to how nerve interaction affected the muscles and eyes (Fishman, 1995). In 1878, Munk identified visual localization in the occipital cortex in dogs and monkeys (Munk, 1890). With the development of neuroscience, neuro-ophthalmology has also developed rapidly. It involves the study of the basis and emergence characteristics of the optic nerve and retina as well as studies on learning, memory, behavior, perception, and consciousness. Therefore, studies of the brain and the eye are always linked closely. Approximately 80% of the external information received by an individual comes from the eyes; this visual information is processed by the visual cortex of the brain (Siu and Murphy, 2018). “Brain” and “eye movement” are keywords that appear frequently; unsurprisingly, the two entities are always closely related in ophthalmology in the field of neuroscience. The mysteries of the brain are being revealed gradually; research on the cerebral cortex is the latest and most advanced in all of the research on the nervous system (Rakic, 2009).

The cortex is implicated in many diseases from depression to schizophrenia, and its pathogenesis is, in part, due to abnormalities in some connections among the involved brain regions. This has led scientists to further focus on these connections. As indicated by burst keywords, functional connectivity and the default mode network (which exhibited the highest overlap with each other in terms of structural and functional connectivity) were defined and gradually brought into focus (Horn et al., 2014). Functional connectivity is the connection among brain regions that share functional properties (Biswal et al., 1997).

The default mode network is a group of interconnected and anatomically defined brain regions; it is mainly reflected in the network of brain regions that continue to perform certain functional activities in the resting state of the human brain in the absence of any tasks (Buckner et al., 2008; Raichle, 2015). Though scholars are currently conducting research on neurodegenerative eye diseases (such as primary open-angle glaucoma and diabetic retinopathy) and these two burst keywords, these efforts are limited (Huang et al., 2020; Wang et al., 2021). However, our findings indicate that the default mode network is the focus of ophthalmic research in neuroscience and will be a hotspot for future researchers. Our bibliometric findings also provide additional future research directions and ideas for further mining of data on vision-related mechanisms.

Neuroscientists worldwide have continued to deepen their research on the brain and in ophthalmology. Scholars in various countries and universities have presented their findings in a number of studies and have had their publications cited in the past 20 years. Babiloni from Italy has published 33 articles in the field; they have majorly conducted experimental studies on clinical electroencephalographic and neuroimaging techniques, focusing on the main progressive neurodegenerative diseases leading to dementia (such as Alzheimer's, Lewy body, and Parkinson's diseases) (Del Percio et al., 2013; Pascarelli et al., 2020; Babiloni et al., 2022). Tononi from the United States has published 21 articles in the field and has ranked first in terms of the total number of citations. They have focused on consciousness and associated disorders as well as on the mechanisms and functions of sleep (Baird et al., 2021, 2022; Valomon et al., 2021). Corbetta has the most extensive references cited in the outbreak period, and their article with the highest outbreak intensity [titled “Control of goal-directed and stimulus-driven attention in the brain” (Corbetta and Shulman, 2002)] has detailed research into two networks of brain areas involved in attention control.

The development of neuro-ophthalmology has gradually been noticed and deepened by scholars. Schall has focused on neuroscience, saccade, eye movement, visual search, and the supplementary eye field. Bisley has focused on vision and visual perception. Surprisingly, although the two scholars have focused on similar research topics, they have not cooperated in any of their studies. As shown in Figure 3, the trend of collaboration among academic researchers in the ophthalmology field of neuroscience is dominated by small groups. Furthermore, the United States dominates research in the field and has made great contributions to it. As shown in Table 2, the most prolific university was the University of Washington, which is located in the United States and has been one of the leading institutions in neuroscience research for many years in the world. The University of Washington is a world-renowned research university with over 150 neuroscience laboratories, which are distributed in the departments of biology, psychology, biological structure, pharmacology, physiology, and biophysics. It provides the neuroscience program with its core instructors. Approximately 70% of neuroscience majors take part in undergraduate research annually. To date, 100% of students in the major who have pursued a research opportunity have successfully found their way into a research laboratory, and such a good academic atmosphere may nourish novel ideas. The University of Washington receives more federal research funding than any other public university in the United States, and many foundations support research projects, such as Brightfocus Foundation and Marfan Foundation. There are special Endowed Research Awards to encourage students to participate in relevant annual conferences, such as Pepose Award: To fund travel to The Association for Research in Vision and Ophthalmology for research in vision and ophthalmology. The University of Washington has trained many well-known scholars, and many scholars have won the Nobel Prize laureates in physiology or medicine for their research, such as Buck won the prize for discoveries of odorant receptors and the organization of the olfactory system. Hartwell won the prize for discoveries of key regulators of the cell cycle. All of those have greatly enhanced the regional advantages and further strengthened the academic influence of the United States in this field, which also partly explains why the United States has always maintained a leading position in this research field. However, related research in China and India remains in its early stages. These countries need to continue to learn from other countries and conduct in-depth research in the field. Therefore, in future, we will encourage global exchanges and cooperation, promote the development of cutting-edge disciplines, further solve important scientific problems, and contribute to the wellbeing of mankind.

In summary, this study contributes valuable information to potential collaborators and institutions by providing insights into the developmental trend of ophthalmology in the field of neuroscience. It also sheds light on new directions for further research.

## 5. Limitations

Being the first bibliometric analysis of neuroscience in ophthalmology, this study has some limitations. First, the keywords were selected from terms that have only appeared in titles, abstracts, and keywords. Due to technical limitations, relevant terms could not be retrieved from full texts for analysis. Moreover, only articles were considered; no reviews, patents, abstracts, and articles were included in our analysis. Second, because retrieval was limited to the Web of Science Core Collection of journals, documents that are not included in this database were not considered. Finally, we only analyzed articles published in English, possibly ignoring some valuable articles in other languages. Nevertheless, we believe that our study can still be used to present the overall situation and trends in the field.

## 6. Conclusion and prospect

In the last 20 years, the number of publications in the field of neuro-ophthalmology has increased gradually. We have conducted a comprehensive analysis of ophthalmology-related literature in neuroscience from two perspectives: global research trends and major contributors. A large body of evidence indicates that the development of neuro-ophthalmology plays a key role in improvement of neuroscience. Overall, our bibliometric study provides important evidence for subsequent researchers to better understand basic knowledge patterns, discover potential opportunities for collaboration with other research teams, identify current research hotspots, and grasp future research frontiers.

## Data availability statement

The original contributions presented in the study are included in the article/supplementary material, further inquiries can be directed to the corresponding author.

## Author contributions

XX and LS conceived the study. XX drafted and edited the manuscript. LL, ZC, QC, TH, YY, and LS contributed to revising the manuscript. All authors contributed to the article and approved the submitted version.

## Footnotes

^1^ https://sourceforge.net/projects/citespace/files/latest/download

^2^ https://www.vosviewer.com/download#downloadvosviewer

^3^ https://www.microsoft.com/zh-cn/microsoft-365/excel

## References

[B1] Babiloni C. Lorenzo I. Lizio R. Lopez S. Tucci F. Ferri R.. (2022). Reactivity of posterior cortical electroencephalographic alpha rhythms during eyes opening in cognitively intact older adults and patients with dementia due to Alzheimer's and Lewy body diseases. Neurobiol. Aging 115, 88–108. 10.1016/j.neurobiolaging.2022.04.001 35512497

[B2] Baird B. LaBerge S. Tononi G. (2021). Two-way communication in lucid REM sleep dreaming. Trends Cogn. Sci. 25, 427–428. 10.1016/j.tics.2021.04.004 33879421

[B3] Baird B. Tononi G. LaBerge S. (2022). Lucid dreaming occurs in activated rapid eye movement sleep, not a mixture of sleep and wakefulness. Sleep 45. 10.1093/sleep/zsab294 35167686

[B4] Bisley J. W. Goldberg M. E. (2003). Neuronal activity in the lateral intraparietal area and spatial attention. Science 299, 81–86. 10.1126/science.1077395 12511644

[B5] Bisley J. W. Goldberg M. E. (2010). Attention, intention, and priority in the parietal lobe. Annu. Rev. Neurosci. 33, 1–21. 10.1146/annurev-neuro-060909-152823 20192813 PMC3683564

[B6] Biswal B. B. Van Kylen J. Hyde J. S. (1997). Simultaneous assessment of flow and BOLD signals in resting-state functional connectivity maps. NMR Biomed. 10, 165–170. 10.1002/(SICI)1099-1492(199706/08)10:4/<165::AID-NBM45>3.0.CO;2-7 9430343

[B7] Boudry C. Mouriaux F. (2015). Eye neoplasms research: a bibliometric analysis from 1966 to 2012. Eur. J. Ophthalmol. 25, 357–365. 10.5301/ejo.5000556 25612654

[B8] Brown G. L. Harvey A. M. (1941). Neuro-muscular transmission in the extrinsic muscles of the eye. J. Physiol. 99, 379–399. 10.1113/jphysiol.1941.sp003910 16995260 PMC1394082

[B9] Buckner R. L. Andrews-Hanna J. R. Schacter D. L. (2008). The brain's default network: anatomy, function, and relevance to disease. Ann. N. Y. Acad. Sci. 1124, 1–38. 10.1196/annals.1440.011 18400922

[B10] Carvalho J. Invernizzi A. Martins J. Renken R. J. Cornelissen F. W. (2022). Local neuroplasticity in adult glaucomatous visual cortex. Sci. Rep. 12, 21981. 10.1038/s41598-022-24709-1 36539453 PMC9767937

[B11] Chai Z. Silverman D. Li G. Williams D. Raviola E. Yau K. W. (2020). Light-dependent photoreceptor orientation in mouse retina. Sci Adv 6, eabe2782. 10.1126/sciadv.abe2782 33328242 PMC7744070

[B12] Chen C. (2006). CiteSpace II: detecting and visualizing emerging trends and transient patterns in scientific literature. J. Assoc. Inf. Sci. Technol. 57, 359–377. 10.1002/asi.20317

[B13] Chen C. (2017). Science mapping: a systematic review of the literature. J. Data Inf. Sci. 2, 1–40. 10.1515/jdis-2017-0006

[B14] Chen C. Song M. (2019). Visualizing a field of research: a methodology of systematic scientometric reviews. PLoS ONE 14, e0223994. 10.1371/journal.pone.0223994 31671124 PMC6822756

[B15] Chen L. Li G. Jiang Z. Yau K. W. (2023). Unusual phototransduction via cross-motif signaling from G(q) to adenylyl cyclase in intrinsically photosensitive retinalganglion cells. Proc. Natl. Acad. Sci. U. S. A. 120, e2216599120. 10.1073/pnas.2216599120 36584299 PMC9910442

[B16] Cherayil N. R. Tamhankar M. A. (2021). Neuro-Ophthalmology for Internists. Med. Clin. N. Am. 105, 511–529. 10.1016/j.mcna.2021.01.005 33926644

[B17] Clark D. Eggenberger E. (2012). Neuro-ophthalmology of movement disorders. Curr. Opin. Ophthalmol. 23, 491–496. 10.1097/ICU.0b013e328358ba14 23014265

[B18] Corbetta M. Patel G. Shulman G. L. (2008). The reorienting system of the human brain: from environment to theory of mind. Neuron 58, 306–324. 10.1016/j.neuron.2008.04.017 18466742 PMC2441869

[B19] Corbetta M. Shulman G. L. (2002). Control of goal-directed and stimulus-driven attention in the brain. Nat. Rev. Neurosci. 3, 201–215. 10.1038/nrn755 11994752

[B20] Cursiefen C. (2019). Ophthalmology: our discipline with a future. Ophthalmologe 116, 815–816. 10.1007/s00347-019-0914-2 31482233

[B21] Del Percio C. Triggiani A. I. Marzano N. Valenzano A. De Rosas M. Petito A.. (2013). Poor desynchronisation of resting-state eyes-open cortical alpha rhythms in obese subjects without eating disorders. Clin. Neurophysiol. 124, 1095–1105. 10.1016/j.clinph.2013.01.001 23433948

[B22] Di Ieva A. Tschabitscher M. Prada F. Gaetani P. Aimar E. Pisano P.. (2007). The neuroanatomical plates of Guido da Vigevano. Neurosurg. Focus 23, E15. 10.3171/FOC-07/07/E15 17961048

[B23] Duong D. K. Leo M. M. Mitchell E. L. (2008). Neuro-ophthalmology. Emerg. Med. Clin. N. Am. 26, 137–180, vii. 10.1016/j.emc.2007.11.004 18249261

[B24] Egghe L. (2006). Theory and practise of the g-index. Scientometrics 69, 131–152. 10.1007/s11192-006-0144-7

[B25] Falagas M. E. Pitsouni E. I. Malietzis G. A. Pappas G. (2008). Comparison of PubMed, scopus, web of science, and google scholar: strengths and weaknesses. FASEB J. 22, 338–342. 10.1096/fj.07-9492LSF 17884971

[B26] Fan X. Markram H. (2019). A brief history of simulation neuroscience. Front. Neuroinform. 13, 32. 10.3389/fninf.2019.00032 31133838 PMC6513977

[B27] Fishman R. S. (1995). Brain wars: passion and conflict in the localization of vision in the brain. Doc. Ophthalmol. 89, 173–184. 10.1007/BF01203410 7555576

[B28] Gross C. G. (2009). History of neuroscience: early neuroscience. Encycl. Neurosci. 1167–1171. 10.1016/B978-008045046-9.00992-X

[B29] Hegde J. (2018). Neural mechanisms of high-level vision. Compr. Physiol. 8, 903–953. 10.1002/cphy.c160035 29978891

[B30] Horn A. Ostwald D. Reisert M. Blankenburg F. (2014). The structural-functional connectome and the default mode network of the human brain. Neuroimage 102 (Pt 1), 142–151. 10.1016/j.neuroimage.2013.09.069 24099851

[B31] Huang X. Tong Y. Qi C. X. Dan H. D. Deng Q. Q. Shen Y. (2020). Large-scale neuronal network dysfunction in diabetic retinopathy. Neural Plast. 2020, 6872508. 10.1155/2020/6872508 32399026 PMC7204201

[B32] Ionescu G. Frey A. Guyader N. Kristensen E. Andreev A. Guérin-Dugué A. (2022). Synchronization of acquisition devices in neuroimaging: an application using co-registration of eye movements and electroencephalography. Behav. Res. Methods 54, 2545–2564. 10.3758/s13428-021-01756-6 34918232

[B33] Kandel E. Schwartz J. Jessell T. Siegelbaum S. Hudspeth A. J. (2013). Principles of Neural Science, 5th Edn. New York, NY: McGraw-Hill.

[B34] Mansour A. M. Mollayess G. E. Habib R. Arabi A. Medawar W. A. (2015). Bibliometric trends in ophthalmology 1997-2009. Indian J. Ophthalmol. 63, 54–58. 10.4103/0301-4738.151471 25686064 PMC4363959

[B35] Mayr P. Scharnhorst A. (2015). Scientometrics and information retrieval: weak-links revitalized. Scientometrics 102, 2193–2199. 10.1007/s,11192-014-1484-3

[B36] Munk H. (1890). Of the visual area of the cerebral cortex, and its relation to eye movements ^*^. Brain 13, 45–70. 10.1093/brain/13.1.45

[B37] Neuro-ophthalmology Group of Ophthalmology Branch of Chinese Medical Association (2022). Chinese expert consensus on diagnosis and treatment of diabetic optic neuropathy (2022). Zhonghua Yan Ke Za Zhi 58, 405–411. 10.3760/cma.j.cn112142-20211221-00593 35692021

[B38] Norcia A. M. (2013). Linking perception to neural activity as measured by visual evoked potentials. Vis. Neurosci. 30, 223–227. 10.1017/S0952523813000205 23879990

[B39] Oydanich M. Schott B. Wagner R. S. Guo S. (2022). Bibliometric analysis of the top 100 cited articles in pediatric ophthalmology. J. Pediatr. Ophthalmol. Strabismus 2022, 1–7. 10.3928/01913913-20220809-03 36102264

[B40] Pascarelli M. T. Del Percio C. De Pandis M. F. Ferri R. Lizio R. Noce G.. (2020). Abnormalities of resting-state EEG in patients with prodromal and overt dementia with Lewy bodies: relation to clinical symptoms. Clin. Neurophysiol. 131, 2716–2731. 10.1016/j.clinph.2020.09.004 33039748

[B41] Raichle M. E. (2015). The brain's default mode network. Annu. Rev. Neurosci. 38, 433–447. 10.1146/annurev-neuro-071013-014030 25938726

[B42] Rakic P. (2009). Evolution of the neocortex: a perspective from developmental biology. Nat. Rev. Neurosci. 10, 724–735. 10.1038/nrn2719 19763105 PMC2913577

[B43] Rampat R. Deshmukh R. Chen X. Ting D. S. W. Said D. G. Dua H. S.. (2021). Artificial intelligence in cornea, refractive surgery, and cataract: basic principles, clinical applications, and future directions. Asia Pac. J. Ophthalmol. 10, 268–281. 10.1097/APO.0000000000000394 34224467 PMC7611495

[B44] Roussy M. Mendoza-Halliday D. Martinez-Trujillo J. C. (2021). Neural substrates of visual perception and working memory: two sides of the same coin or two different coins? Front. Neural Circ. 15, 764177. 10.3389/fncir,.2021.764177 34899197 PMC8662382

[B45] Schultz S. K. (2001). Principles of neural science, 4th ed. Am. J. Psychiatry 158, 662–662. 10.1176/appi.ajp.158.4.662

[B46] Shulman R. G. (2013). Brain Imaging: What it Can (and Cannot) Tell Us About Consciousness. New York, NY: Oxford Academic.

[B47] Siu C. R. Murphy K. M. (2018). The development of human visual cortex and clinical implications. Eye Brain 10, 25–36. 10.2147/EB.S130893 29760575 PMC5937627

[B48] Spiegel S. J. Moss H. E. (2021). Neuro-ophthalmic emergencies. Neurol. Clin. 39, 631–647. 10.1016/j.ncl.2021.01.004 33896536 PMC8081067

[B49] Tan Y. Zhu W. Zou Y. Zhang B. Yu Y. Li W.. (2022). Hotspots and trends in ophthalmology in recent 5 years: Bibliometric analysis in 2017-2021. Front. Med. 9, 988133. 10.3389/fmed.2022.988133 36091704 PMC9462464

[B50] Valomon A. Riedner B. A. Jones S. G. Nakamura K. P. Tononi G. Plante D. T.. (2021). A high-density electroencephalography study reveals abnormal sleep homeostasis in patients with rapid eye movement sleep behavior disorder. Sci. Rep. 11, 4758. 10.1038/s41598-021-83980-w 33637812 PMC7910582

[B51] van Eck N. J. Waltman L. (2010). Software survey: VOSviewer, a computer program for bibliometric mapping. Scientometrics 84, 523–538. 10.1007/s11192-009-0146-3 20585380 PMC2883932

[B52] Waltman L. Van Eck N. J. Noyons E. C. M. (2010). A unified approach to mapping and clustering of bibliometric networks. J. Informetr. 4, 629–635. 10.1016/j.joi.2010.07.002

[B53] Wan H. Yan Y. D. Hu X. M. Shang L. Chen Y. H. Huang Y. X.. (2023). Inhibition of mitochondrial VDAC1 oligomerization alleviates apoptosis and necroptosis of retinal neurons following OGD/R injury. Ann. Anat. 247, 152049. 10.1016/j.aanat.2023.152049 36690044

[B54] Wang Q. Qu X. Chen W. Wang H. Huang C. Li T.. (2021). Altered coupling of cerebral blood flow and functional connectivity strength in visual and higher order cognitive cortices in primary open angle glaucoma. J. Cereb. Blood Flow Metab. 41, 901–913. 10.1177/0271678X20935274 32580669 PMC7983497

[B55] Wei S. (2014). Work hard to develop neuro-ophthalmology in China. Zhonghua Yan Ke Za Zhi 50, 881–885. 10.3760/cma.j.issn.0412-4081.2014.12.001 25619177

[B56] Wei S. H. Song H. L. Tong Y. (2020). The development history and prospect of neuro-ophthalmology in China. Zhonghua Yan Ke Za Zhi 56, 891–894. 10.3760/cma.j.cn112142-20200602-00369 33342115

[B57] Yan W. T. Zhao W. J. Hu X. M. Ban X. X. Ning W. Y. Wan H.. (2023). PANoptosis-like cell death in ischemia/reperfusion injury of retinal neurons. Neural Regen Res 18, 357–363. 10.4103/1673-5374.346545 35900430 PMC9396479

[B58] Yao H. Lu H. Wang W. (2010). Visual neuroscience research in China. Sci. China Life Sci. 53, 363–373. 10.1007/s11427-010-0071-y 20596932

[B59] Zhang Q. Hu X. M. Zhao W. J. Ban X. X. Li Y. Huang Y. X.. (2023). Targeting necroptosis: a novel therapeutic option for retinal degenerative diseases. Int. J. Biol. Sci. 19, 658–674. 10.7150/ijbs.77994 36632450 PMC9830514

[B60] Zhou Z. Shang L. Zhang Q. Hu X. Huang J. F. Xiong K. (2023). DTX3L induced NLRP3 ubiquitination inhibit R28 cell pyroptosis in OGD/R injury. Biochim. Biophys. Acta Mol. Cell. Res. 1870, 119433. 10.1016/j.bbamcr.2023.119433 36706922

[B61] Zhu X. Hu J. Deng S. Tan Y. Qiu C. Zhang M.. (2020). Bibliometric and visual analysis of research on the links between the gut microbiota and depression from 1999 to 2019. Front. Psychiatry 11, 587670. 10.3389/fpsyt.2020.587670 33488420 PMC7819979

